# Supplementary material for: Revealing nano-scale lattice distortions in implanted material with 3D Bragg ptychography
Source: Nat Commun. 2021 Dec 3;12:7059. doi: 10.1038/s41467-021-27224-5 (PMC8642407; doi:10.1038/s41467-021-27224-5)
Supplement: Supplementary file 1 — Supplementary Information [file 41467_2021_27224_MOESM1_ESM.pdf]

Supplementary information for

Revealing nano-scale lattice distortions in implanted material with 3D Bragg ptychography

Peng Li[1,+], Nicholas W. Phillips[2, ++], Steven Leake[3], Marc Allain[1], Felix Hofmann[2], Virginie Chamard[1,*]

[1] Aix-Marseille Univ, CNRS, Centrale Marseille, Institut Fresnel, Marseille, France.

[2] Department of Engineering Science, University of Oxford, Parks Road, Oxford, OX1 3PJ, United Kingdom.

[3] ESRF – The European Synchrotron, 71 Avenue des Martyrs, 38000 Grenoble, France.

[+] Now at Diamond Light Source, Harwell Science and Innovation Campus, Fermi Ave, Didcot OX11 0DE, United Kingdom.

[++] Now at Paul Scherrer Institut, 5232 Villigen PSI, Switzerland.

*Correspondence to: V. Chamard, virginie.chamard@fresnel.fr

This document contains

- Supplementary Figures 1-11

Figure 1 – Experimental data-set
Figure 2 – Reconstruction with other probe initial estimates
Figure 3 – Definition of the mask used for plotting the retrieved object
Figure 4 – 3D resolution estimation
Figure 5 – 3D unwrapped phase map of the object
Figure 6 – Complementary approaches (EBSD and micro-Laue diffraction)
Figure 7 – Limit of the up-sampling approach
Figure 8 - Calculated implantation and damage profiles: Comparison with experiment
Figure 9 – Strain profile at the grain boundary
Figure 10 – Dislocation phase field analysis
Figure 11 – 3D strain and tilts

- Supplementary Table 1

Table 1 – Ion energies and fluences

Fig. 1 - Experimental data-set. (a) Complete rocking curves for both unimplanted and implanted regions, measured prior to the Bragg ptychography acquisition. The gray rectangle indicates the angular range chosen for the Bragg ptychography measurements. (b) The rocking curves at positions #381 and #400 extracted from the Bragg ptychography data-set. Note that the complete rocking curves were not taken at exactly the same sample positions as #381 and #400 from the Bragg ptychography measurements. (c) 3D rendering of the intensity distribution (iso-surface in green) obtained at position #381 during the Bragg ptychography acquisition and plotted in an orthogonal frame. One detector plane position is highlighted by a dashed rectangle. (d) 2D intensity distribution shown in the plane highlighted in (c). (e) and (f) same as (c) and (d), respectively, for position #400. For (d) and (f), the intensity color scale is indicated on the plots.

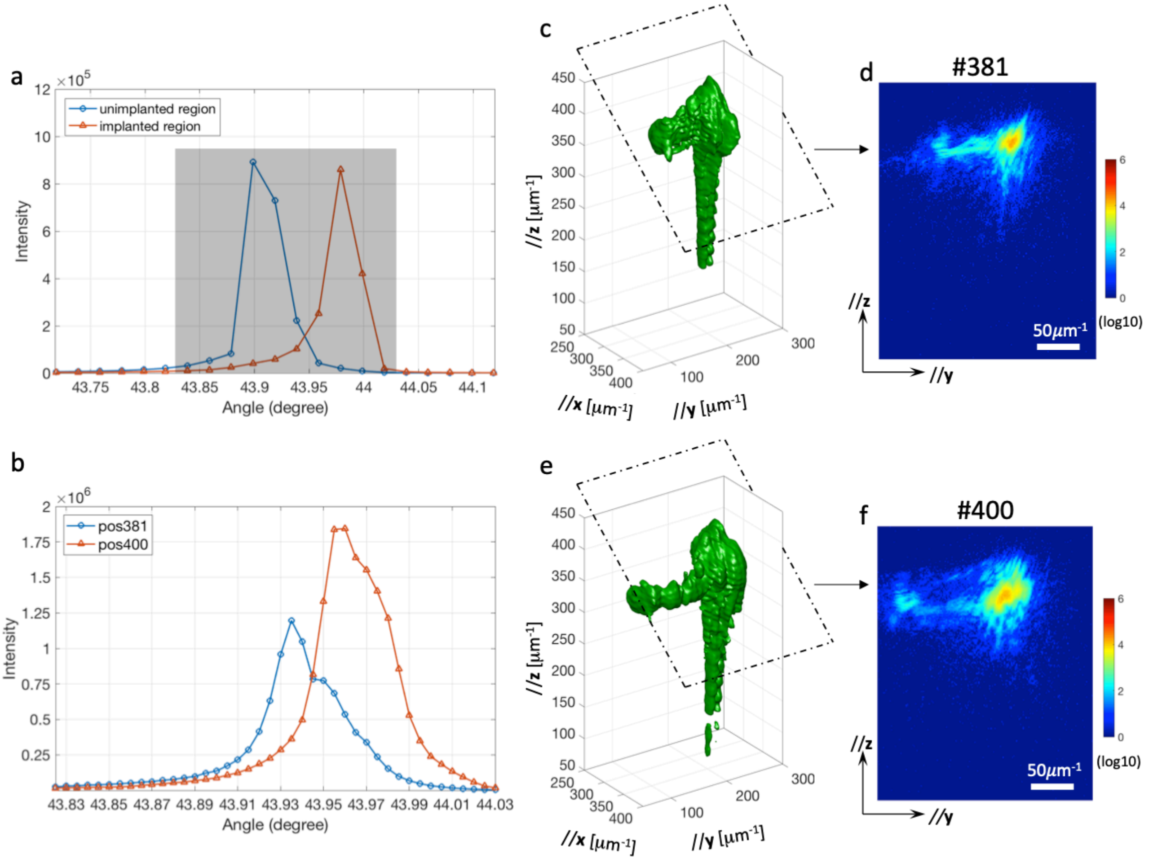

Fig. 2 - Reconstructions with other initial probe estimates. For each column, we present probe and object reconstruction (probe in hue rendering in the left column and object as amplitude and phase maps, in the middle and right columns, respectively), from the same slice as the one plotted in Fig. 3. (a-c) Results obtained using a Gaussian beam as the initial guess of the probe. Inset is the Gaussian-beam starting estimate. The FWHM of the Gaussian intensity profile is 50% bigger than that of the characterized probe. (d-f) Results obtained using a calculated beam as the probe initial guess. The beam is calculated via a Fourier transform of a rectangular pinhole, whose size is 50% bigger than the numerical aperture of experimental setup along both horizontal and vertical directions. The inset is the calculated beam started estimate. Similar final probe and object reconstructions are obtained, irrespective of the probe start guess. All complex-valued maps are based on the color-map indicated by the color wheel. Color scale for density (in arb. units) and phase (in rad.) are indicated on the plots. The scale bar represents 1 μm.

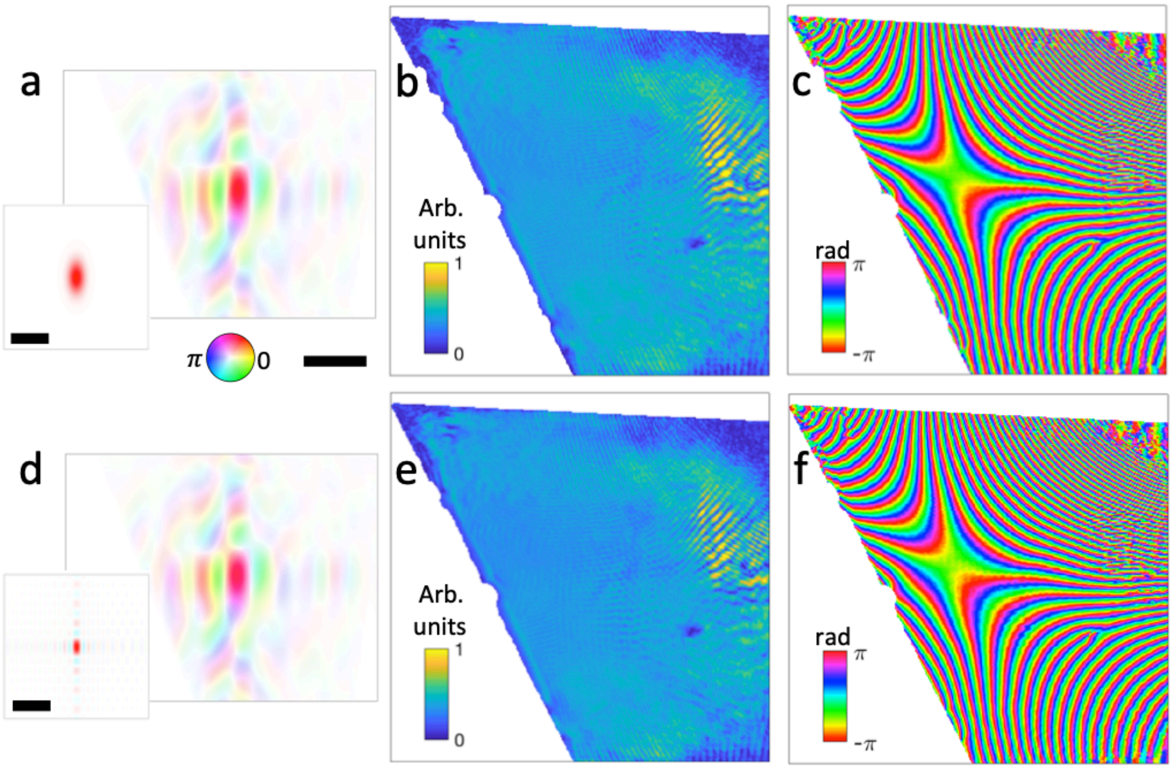

Figure 3 – Definition of the mask used for plotting the retrieved object.

Producing a mask

For sake of clarity, only the relevant parts of the retrieved objects are shown in the figures. To this aim, a Boolean mask is defined and applied to all retrieved maps in the article. To start with, we plot the amplitude of the central data slice of the full object reconstruction frame, for the new angular up-sampled reconstruction and the formerly employed reconstruction (a, b). In addition, to some weak contributions in regions obviously outside the investigated crystalline grain, we observe a striking discrepancy in the apparent location of the top surface of the sample readily identifiable and highlighted by the white lines. Furthermore, a band of high amplitude resides below the interface position in (b). We attribute this to the incomplete recovery of the object, which is adversely affected by the lack of probe refinement. This is further confirmed in (c), which presents the object retrieved with the formerly employed angular sampling rate with probe update. Of particular note here is the correct retrieval of the top surface when the updated probe is used.

These amplitude cross-sections were then used to produce the associated masks, shown as a slice through the centre of the sample volume in (d) and (e). The mask is a composite matrix: the green part corresponds to the area of the sample, where the amplitude is above 0.14. The red regions are manually defined based on the position of the grain boundary and the top surface of the sample. These are used to exclude some erroneous regions, which are not removed completely via an amplitude threshold alone. Their positions are well known from SEM analysis and from the obvious edge features in the recovered amplitude. Finally, the top and bottom surfaces of the highly strained FIB damage layer is further used to enforce a phase gradient based support. For information, an approximately circular region, shown in blue in (d), maps the photon distribution with a threshold of 0.005 % of the maximum intensity (regions reconstructed beyond this may be more likely to contain artefacts). Finally, it is particularly worth noting the substantially enlarged field of view afforded by the up-sampling approach.

Figure caption

Amplitude of the central data slice of the full object reconstruction frame for (a) the new angular up-sampled reconstruction with probe update and (b) the formerly employed angular sampling rate without probe update. The edge of the top surface is marked with white lines. In (a), the two extreme positions of the surface are marked with orange (top) and red (bottom) dotted lines, as guides to the eye. (c) Object retrieved with the formerly employed angular sampling rate with probe update. (d) Mask decomposition used for all angular up-sampled reconstructions (see text above for details). (e) Same as (d) for all reconstructions using the formerly employed angular sampling. In (d), the black rectangle indicates the full reconstruction frame of the object and the region enclosed by the black dashed lines is the region used for the object displays in the article. In (a-c), the used color scale is the same as the one used in Supplementary Figure 2b. In (d, e) the green and red regions refer to the inside and outside of the probed crystal, respectively. The grey areas indicate the regions that received enough photons to be retrieved with confidence. The scale bar is indicated on (c) and is common to all plots.

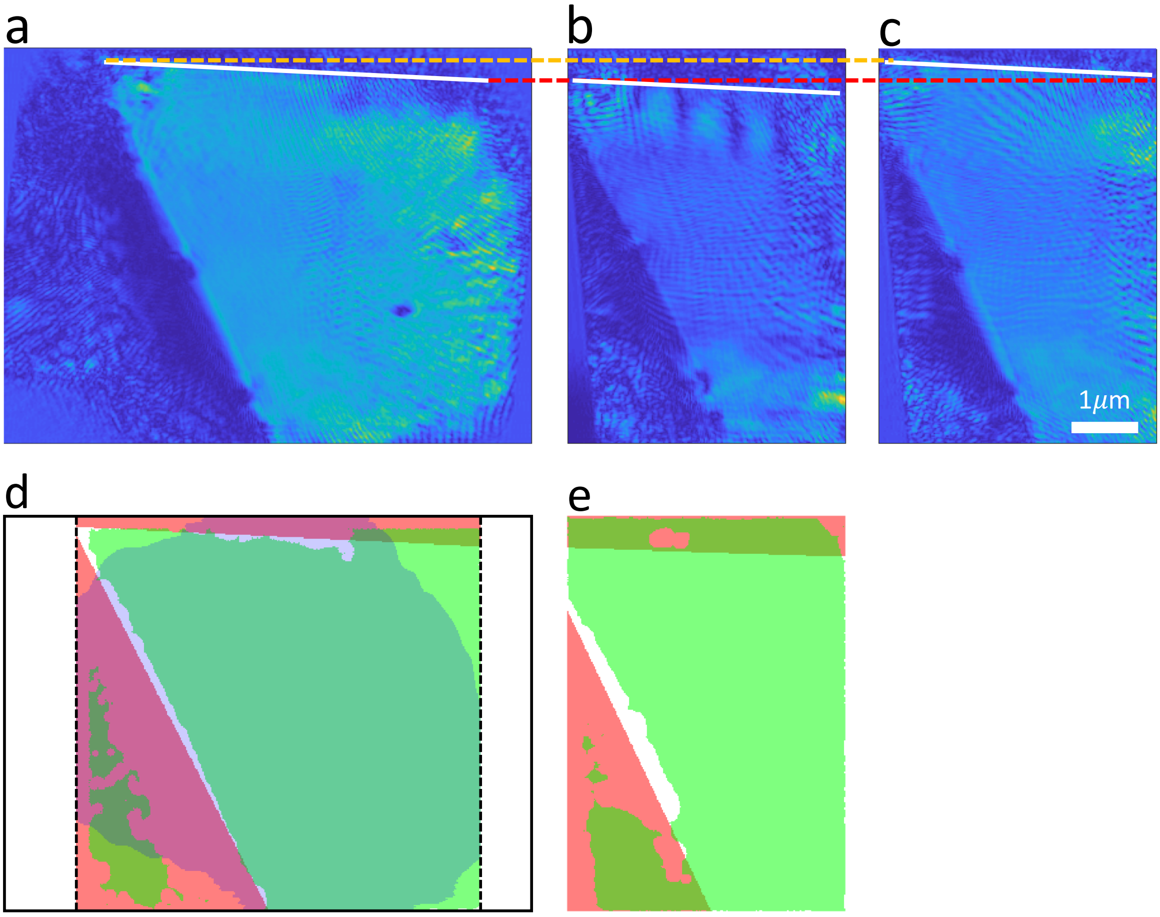

a
b
c
1μm
d
e

Figure 4 – 3D resolution estimation:

Methodology

The Fourier shell correlation approach was used to estimate a lower bound to the 3D resolution[1]. To this aim, the full data-set was separated in two parts. The individual 3D data-set being identified by a pair of numbers representing the spatial translation order, each sub-set of ptychography data was obtained by selecting 3D data-sets with translation numbers of the same parity (*i.e.*, odd/odd and even/even for one subset versus odd/even and even/odd for the second subset). These two data sets were used to produce two reconstructions, from which the 3D Fourier shell correlation curves were calculated. The decay of the curves below a 1/2-bit information threshold was used to define the cut-off spatial frequency f, from which the spatial resolution was derived (given by $1/f$). The overall resolution was estimated to about 48 nm (a). To account for the directional dependence of the spatial resolution, the Fourier shell correlation directional curves were also calculated. They indicate resolutions of 40 nm and 39 nm along **y** (c) and **x** (d), respectively.

Along **z**, this approach indicates an extremely high (non-physical) resolution, as a result of the use of the film support, which introduces sharp edges at the film interface (b). In order to estimate the resolution along **z**, the object amplitude map was used and analyzed in regions where the support is not cropping the object (e). To estimate the resolution along **z**, the **z**-derivative was calculated and the width of the interface transition was estimate from a series of one-dimensional cross sections (e). They indicate a resolution of about 37 nm.

Figure caption

(a) Fourier shell correlation (FSC) curve averaged over all directions. (b-d) Fourier shell correlation curves along the **z**, **y** and **x** directions, respectively. (e) Object amplitude map used to refine the resolution along **z**, shown in the (**x**, **y**) and (**z**, **y**) planes. The plane on the right is the **z**-derivative map. The 1D plots are cross-sections used to estimate the **z**-resolution. The widths of the interfacial transition are indicated on the plots. The color scale used for (e) is the same as the one used in Supplementary Figure 2(b).

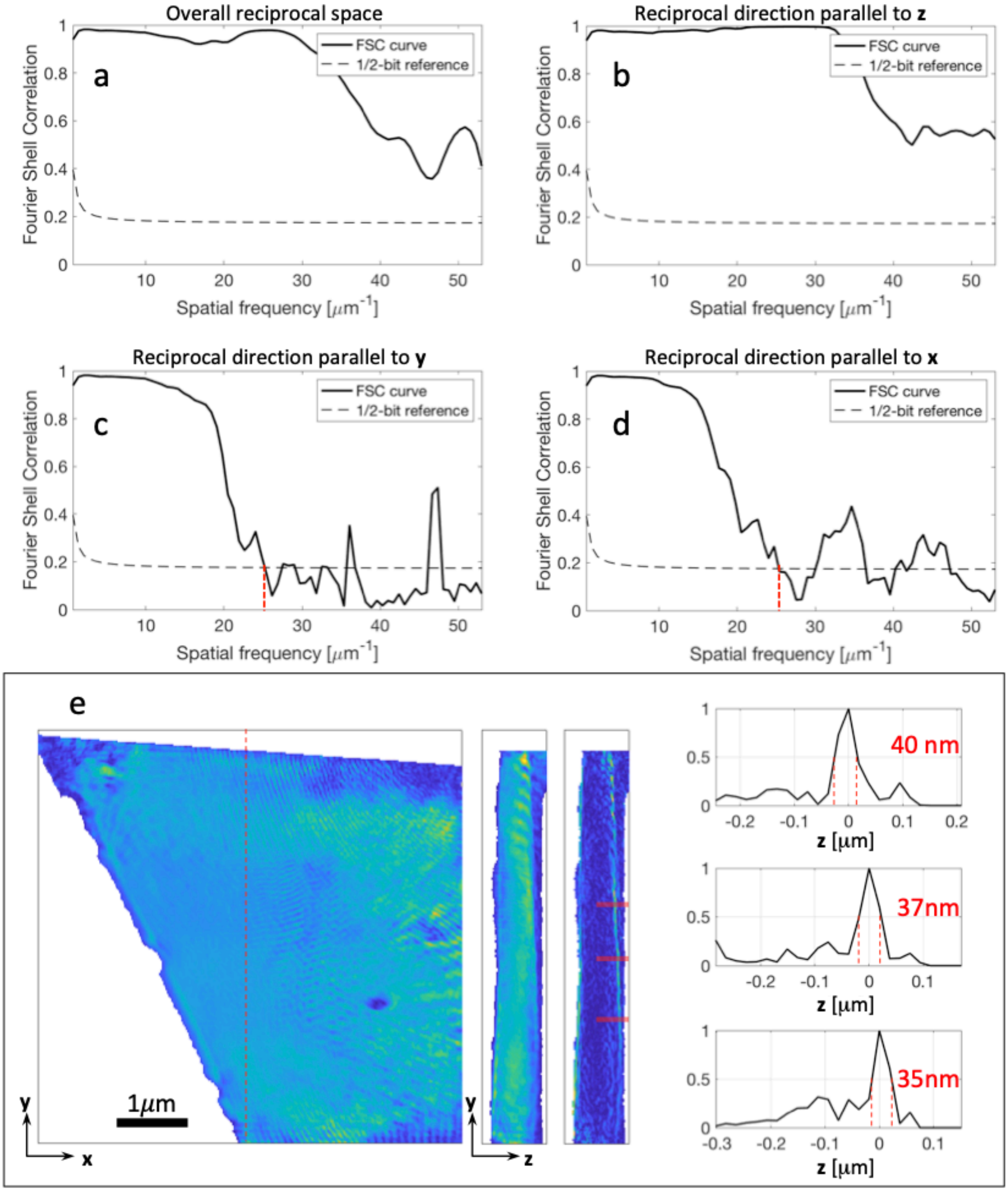
Overall reciprocal space
Reciprocal direction parallel to z
Reciprocal direction parallel to y
Reciprocal direction parallel to x
a
b
c
d
e
FSC curve
1/2-bit reference
Fourier Shell Correlation
Spatial frequency [μm^-1]
1μm
y
x
z
z [μm]
40 nm
37nm
35nm

Figure 5: 3D displacement extracted from the ϕ_{220} phase map of the object. (a) 3D representation of the $\phi_{220}(\mathbf{r})$ phase map as shown in Fig. 3b of the main text. (b) Unwrapped phase converted to a displacement field u($\mathbf{r}$), using $\phi_{220}(\mathbf{r}) = \mathbf{Q}_{220} \cdot \mathbf{u}(\mathbf{r})$. For sake of clarity, the displacement is shown (top) in a series of nine (**z**, **y**) planes taken along the **x** direction and (bottom) in a (**x**, **z**) plane taken at single position along **y**. The color scale in (a) is the same as the one used in Supplementary Figure 2(c). The color scale for (b) and (c) is indicated on the plot.

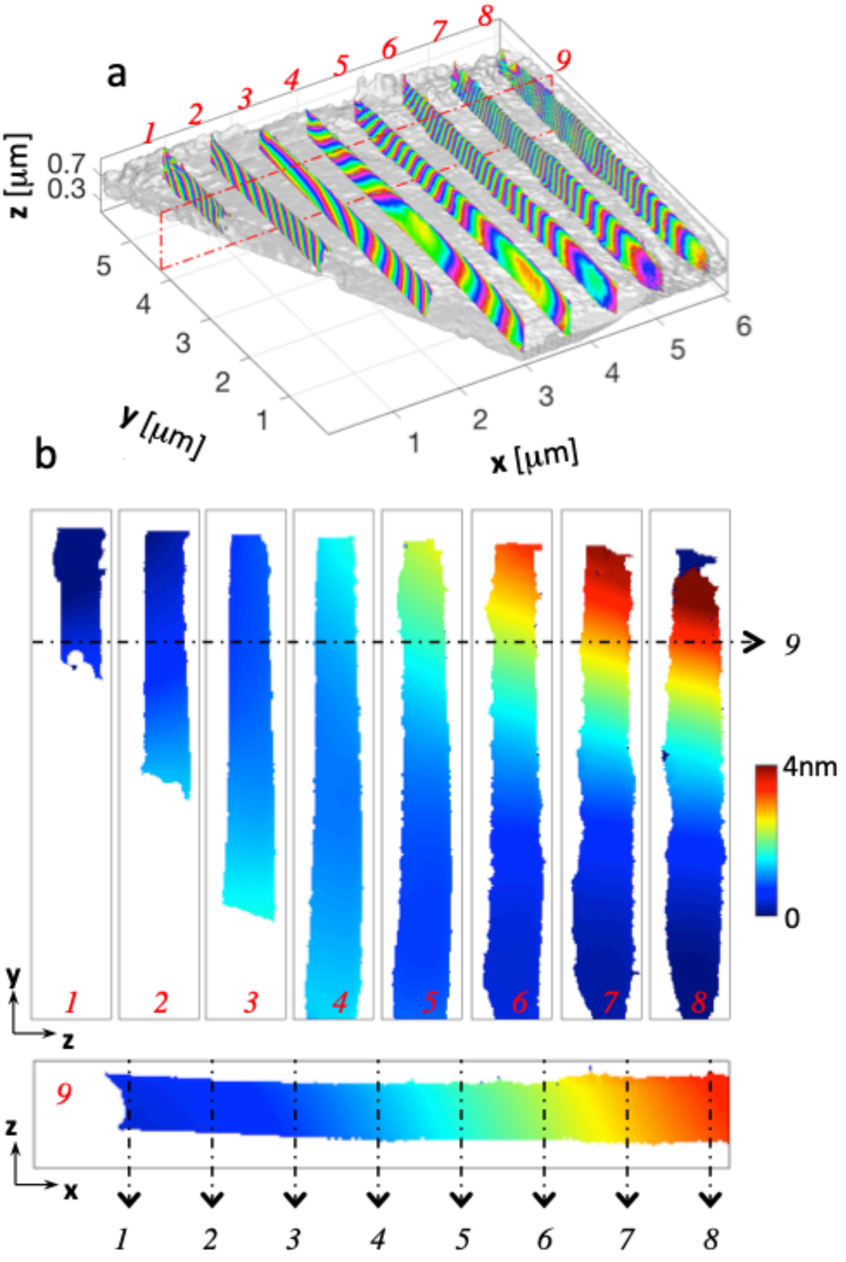

Figure 6: Complementary approaches: High-resolution Electron back scattering diffraction (HR-EBSD) and x-ray micro-beam Laue x-ray diffraction

Experimental details

1 - High-resolution Electron back scattering diffraction: Additional analysis of the sample by HR-EBSD provides high-resolution maps of the deviatoric strain tensor and lattice rotation, to be compared with the BP results. Data were collected on a Zeiss Merlin scanning electron microscope equipped with a Bruker eFlash detector, using an accelerating voltage of 20 keV and specimen tilt of 70 degrees. A step size of 254 nm as used to cover 41×62 (H×V) points over the sample region. Data were analysed using the cross-correlation approach in MATLAB-based HR-EBSD software.[2] To allow for relevant comparison with the BP results, the depth contribution profile in tungsten was simulated for 20 keV, 70° tilt, 2 nm radius beam and 10^6 electron trajectories using Monte Carlo simulation of electron trajectory in solids (Casino software).[3] It was found that one pixel along the **z** direction in the BP reconstruction map (*i. e.*, 19 nm) corresponds to 83.2 % of the total measured signal for HR-EBSD. This single pixel integration depth is later used in the comparison of PB data with HR-EBSD data.

2 - X-ray micro-beam Laue x-ray diffraction: Further comparison with mainstay techniques includes x-ray micro-beam Laue diffraction. This was performed at 34IDE of the Advanced Photon Source, where data were collected by step scanning a white-beam probe (of approximately 300 nm FWHM in the vertical and horizontal directions) over the sample in 500 nm steps. Analysis was performed using LaueGo. Micro-beam Laue diffraction is exceptionally sensitive to strain, with sensitivities of 10^{-4} routinely achieved.[4–6] However, the spatial resolution is limited by the size of the probe in the scanning plane and depth resolution, whilst attainable through the use of a differential aperture,[7,8] is typically of the order of 500 nm to 1 μm. The data presented here being obtained without the differential aperture mode, it is therefore a projection of the strain throughout the thickness of the sample. This was accounted for in the presented comparison with BP.

Comparison with BP results

In order to compare the information brought by BP with the above selected methods, the 3D BP maps were numerically integrated along the depth according to the respective specificities of the two complementary approaches (as described above). The 2D strain and lattice rotation maps obtained with HR-EBSD and micro-beam Laue diffraction are shown in Supplementary Figure 4 together with the corresponding BP-based maps. As expected, the mimicking results from Bragg ptychography still match well with the results from the other two methods, but present much higher resolution and better quality. From the strain map of HR-EBSD diffraction, we cannot see the implantation-induced strain at all, whilst it is poorly resolved in the micro-beam Laue diffraction data. This is likely caused by the integration of the strong FIB-induced strain for the two reference methods. The mimicking BP-based results presents also a strongly reduced strain contrast between implanted and non-implanted layers although the strain is still visible from the micro-beam Laue diffraction mimicking map.

Figure caption

(a1-a3) Micro-Laue (μ-Laue) measurements and (b1-b3) its mimicking results from Bragg ptychography. (c1-c3) HR-EBSD measurements and (d1–d3) its mimicking results from Bragg ptychography. The indices 1, 2, and 3 corresponds to ε_{zz}, ω_y and ω_x, respectively. The red rectangles in (a1-a3) and (c1-c3) indicate the region imaged by Bragg ptychography. Color scales for strain and lattice rotations are indicated on the plots. The lattice rotation values are given in radian. The scale bars represent 1 μm.

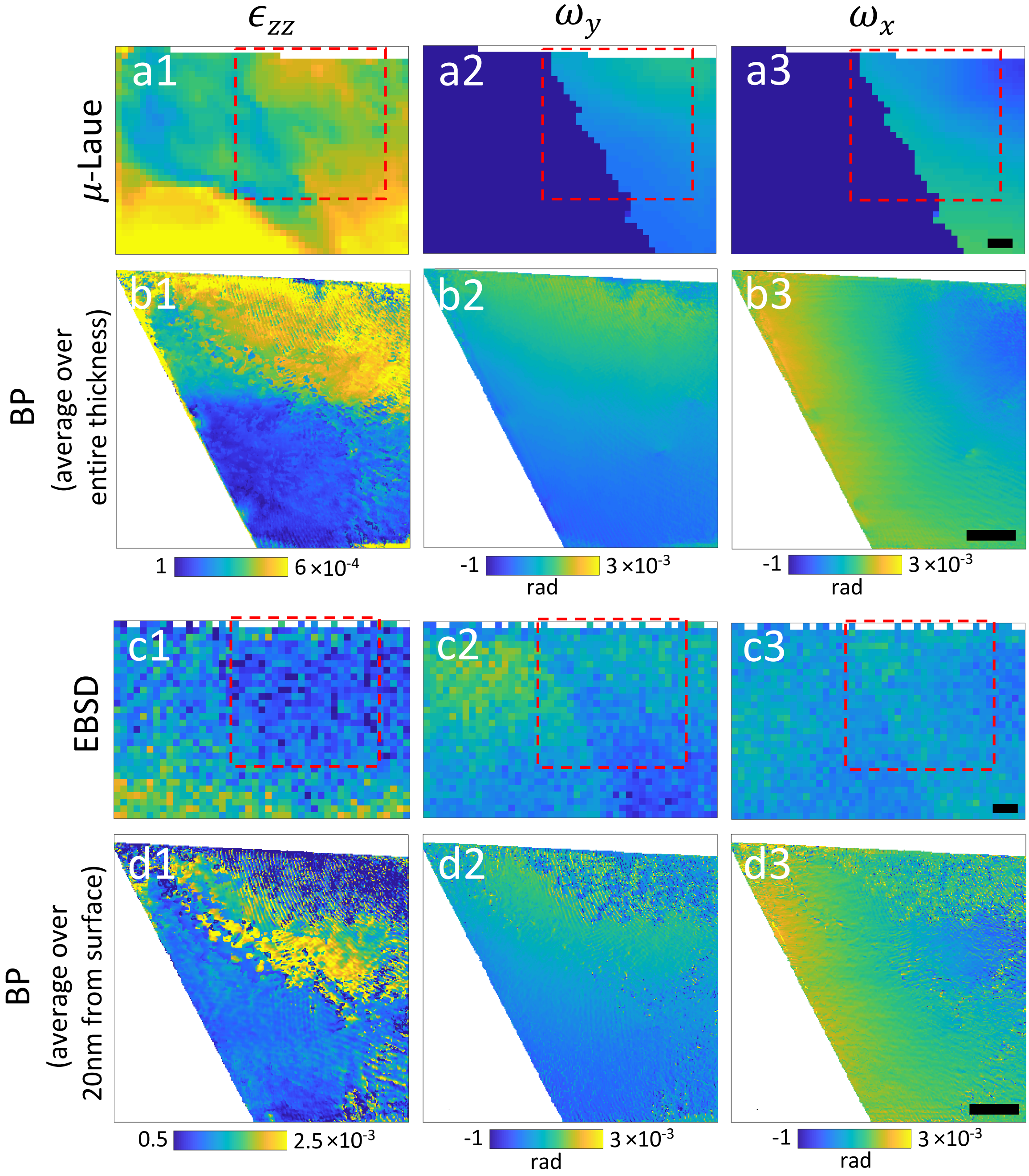

ϵ_{zz}
ω_y
ω_x
μ-Laue
a1
a2
a3
BP
(average over
entire thickness)
b1
b2
b3
1
6 ×10^-4
-1
3 ×10^-3
rad
-1
3 ×10^-3
rad
EBSD
c1
c2
c3
BP
(average over
20nm from surface)
d1
d2
d3
0.5
2.5 ×10^-3
-1
3 ×10^-3
rad
-1
3 ×10^-3
rad

Figure 7 – Limit of the up-sampling approach.

Methodology

To numerically estimate the limit of the up-sampling approach, we performed a series of reconstructions using different up-sampling (u. s.) ratios, while keeping the effective angular step size fixed at 0.0017°. The u. s. ratio is defined as the actual angular step $\Delta\theta$ divided by the effective angular step. For this analysis, we increased the u. s. ratio of the original data along the angular dimension, *i.e.*, used less angular measurements (or bigger angular steps $\Delta\theta$ between two measurements). When the up-sampling ratio increases (*i.e.*, fewer angular measurements), the reconstruction quality decreases until it completely fails for 5 angular measurements (up-sampling ratio of 27 and angular step of 0.045°). Considering the specificities of our experimental set-up, the ideally ultimate angular sampling, given by twice the numerical aperture of the focusing mirrors along the vertical direction (3.7×10^{-4}), should be around 0.042°. Our empirically determined limit (about 0.04°) is slightly smaller, likely as a consequence of the Poisson noise present in the signal and other sources of errors (drifts and/or instabilities from the beamline, etc.). Note that, for u. s. ratio higher than 15, the probe reconstruction needed to be fixed during the first 50 iterations.

Figure caption

Series of reconstructions showing the phase maps of the retrieved object in the (**x**, **y**) plane obtained with different u. s. conditions. The u. s. ratio (u. s. r.), the number of angular measurements and the angular step size are indicated at the top of the plots, for each reconstruction. The color scale is indicated on the plot.

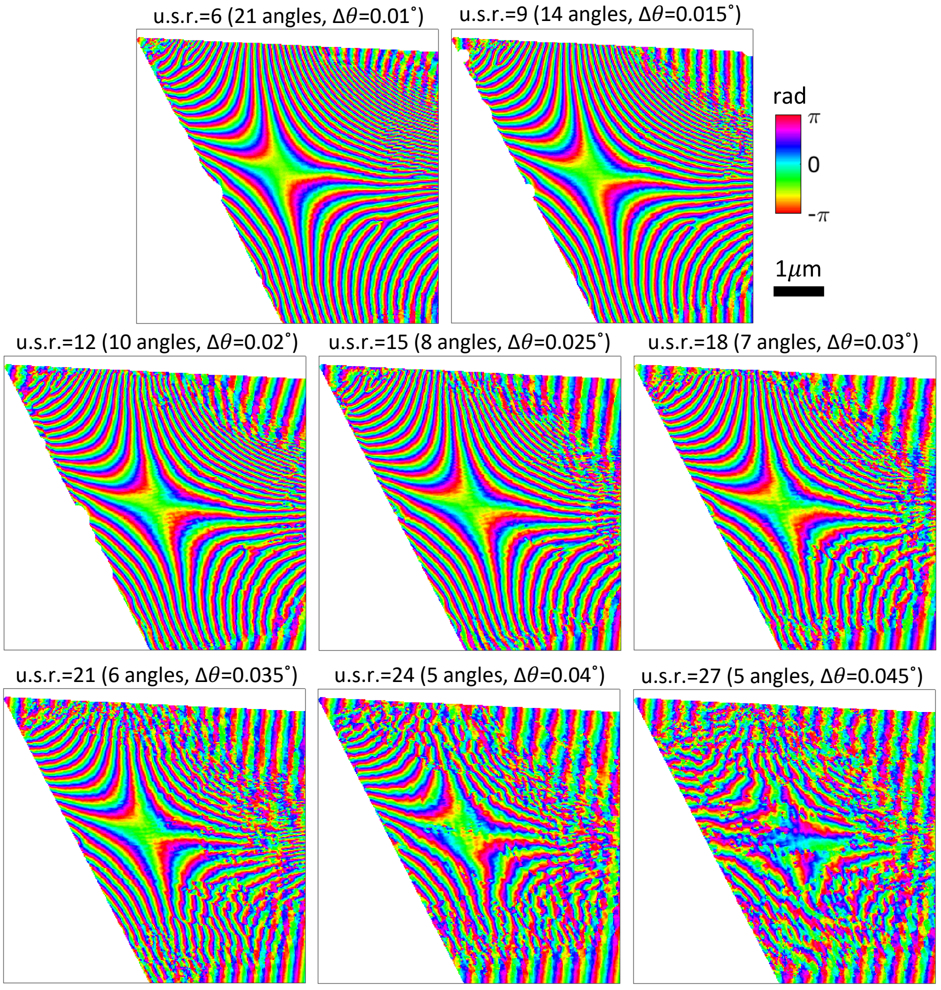

Figure 8 – Calculated implantation and damage profiles: Comparison with experiment

Simulation details

As a starting point, the injected He concentration is calculated by SRIM,[16] together with the induced damage distribution, both of them being extracted from Das *et al.*[17] While the implantation profile shows a number of distinct peaks, the damage distribution is actually quite uniform (See figure below). Hence, a reasonably uniform distribution of the associated lattice strains is expected. This is because vacancies are effectively immobile at the present implantation conditions.[18–20] He in interstitial form, on the other hand, is rather mobile by comparison. As such, one would expect He to move around the lattice until it finds a vacancy to get bound to. Previous theoretical work indicated that the resulting complexes, consisting of He-filled V with SIAs bound to them in close vicinity are rather stable.[21]

In order to compare the experimentally measured strain in the sample as a function of position along the implantation direction, and the strain anticipated based on SRIM calculations, the following models were considered: the damage microstructure was approximated as either consisting of Frenkel pairs where the vacancy is filled with 1 He atom (equation 1), or as Frenkel pairs alone (equation 2).

$$\varepsilon_{zz} = 1/3 * (\Omega_r(SIA) + \Omega_r(V+He)) * damage*recombination \quad (1)$$

$$\varepsilon_{zz} = 1/3 * (\Omega_r(SIA) + \Omega_r(V)) * damage*recombination \quad (2)$$

'damage' here refers to the damage at a particular depth in the sample. The only free parameter is 'recombination', a fixed constant that described the proportion of the SRIM-predicted damage that is retained in the material. It is worth noting that this expression or strain is different to that provided in Hofmann *et al.*[14] The reason is that in the present case the sample preparation has removed the lateral constraints incorporated in the expression in Hofmann *et al.*

The plot shown below was obtained for a constant recombination value of 3.8%. That is of the ~66 Frenkel pairs generated per injected He, as predicted by SRIM, only ~2.5 Frenkel pairs per He are retained. As such we have a He : Frenkel pair ratio of 1:2.5, which justifies the two limits considered in equations (1) and (2). The plot below shows these two limits, using the same recombination value of 3.8%. The agreement with the experimentally measured strain profile is remarkably good between depths of 0.5 and 3.5 microns.

Figure Caption

He implantation profile (green) and resulting strain profiles assuming that the damage microstructure is either consisting of Frenkel pairs where the vacancy is filled with 1 He atom (eq. 1, red, noted as SIA + VHe) or as Frenkel pairs alone (eq. 2, blue, noted as SIA + V). Those calculated strain profiles are compared to the experimental strain profile, extracted along the implantation direction (black). The overall amplitude, shape and finer details are in good agreement with the models. Note that the grey area corresponds to a region where aliasing is locally degrading the retrieved strain profile. The arrows correspond to the relevant axis for each plot. Further details on the calculations are provided above.

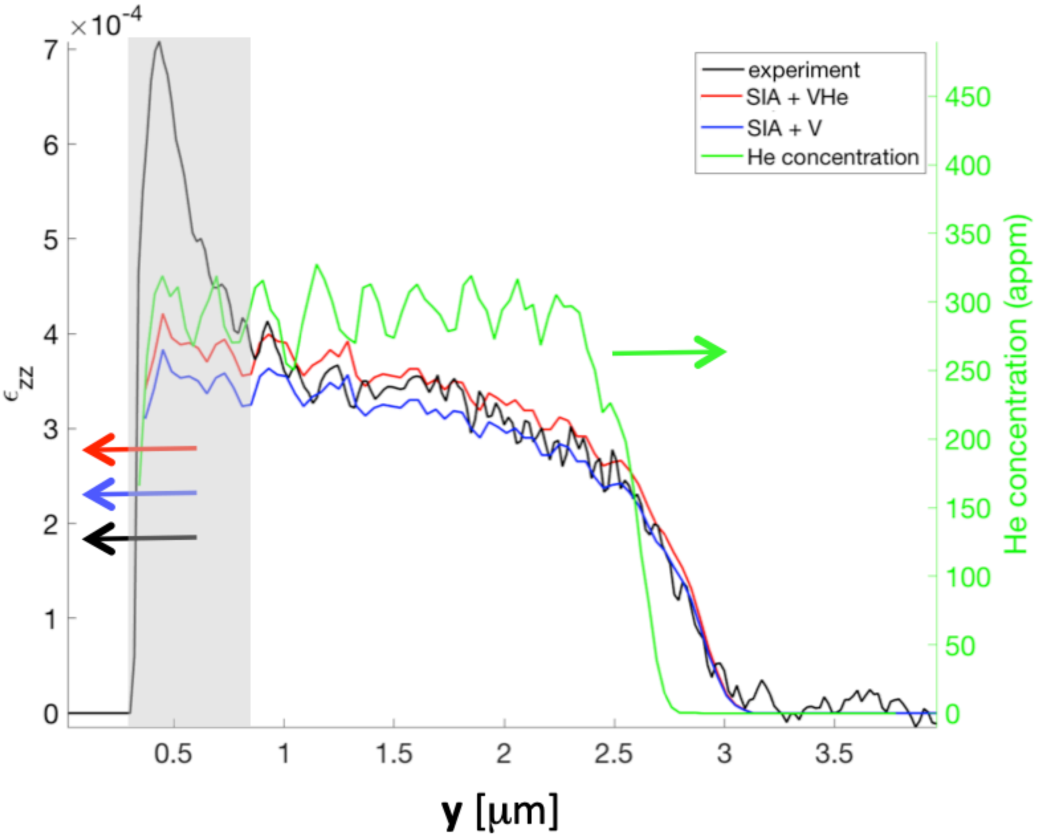

×10^-4
0
1
2
3
4
5
6
7
ϵ_{zz}
0.5
1
1.5
2
2.5
3
3.5
y [μm]
experiment
SIA + VHe
SIA + V
He concentration
0
50
100
150
200
250
300
350
400
450
He concentration (appm)

Figure 9 – Strain profile at the grain boundary. (a) 2D strain map, exhibiting the implanted and non-implanted crystalline parts. The different areas, chosen to investigate the presence of the denuded region near the grain boundary, are shown as colored regions and labels. The color scale is indicated on the plot. (b) One-dimensional strain profile extracted across the grain boundary, in the implanted and non-implanted parts, averaged over the grey regions in (a). Those regions have been selected because they do not contain any visible dislocation and dislocation-associated strain fields. Note that for comparison purposes, the strain profiles have been vertically shifted so that the strain values match the one extracted from the implanted layer. (c) Detailed analysis of the strain profile in the implanted region at the grain boundary. The orange dots represent the one-dimensional experimental strain profile averaged over 8 pixels. The error bars represent the standard deviations. The three lines are fits of the data considering a spatial resolution of 39 nm and assuming (*i*) no denuded region, (*ii*) a fully defect-denuded region (57 nm wide) and (*iii*) a partially defect-denuded region (strain of 2.08×10^{-4} and width of 70 nm). Only the partially defect-denuded region model is able to account for the strain variation at the boundary. (d) One-dimensional strain profiles, extracted along the grain boundary at two different distances from the boundary. A faint decay of the strain is observed in the implanted region.

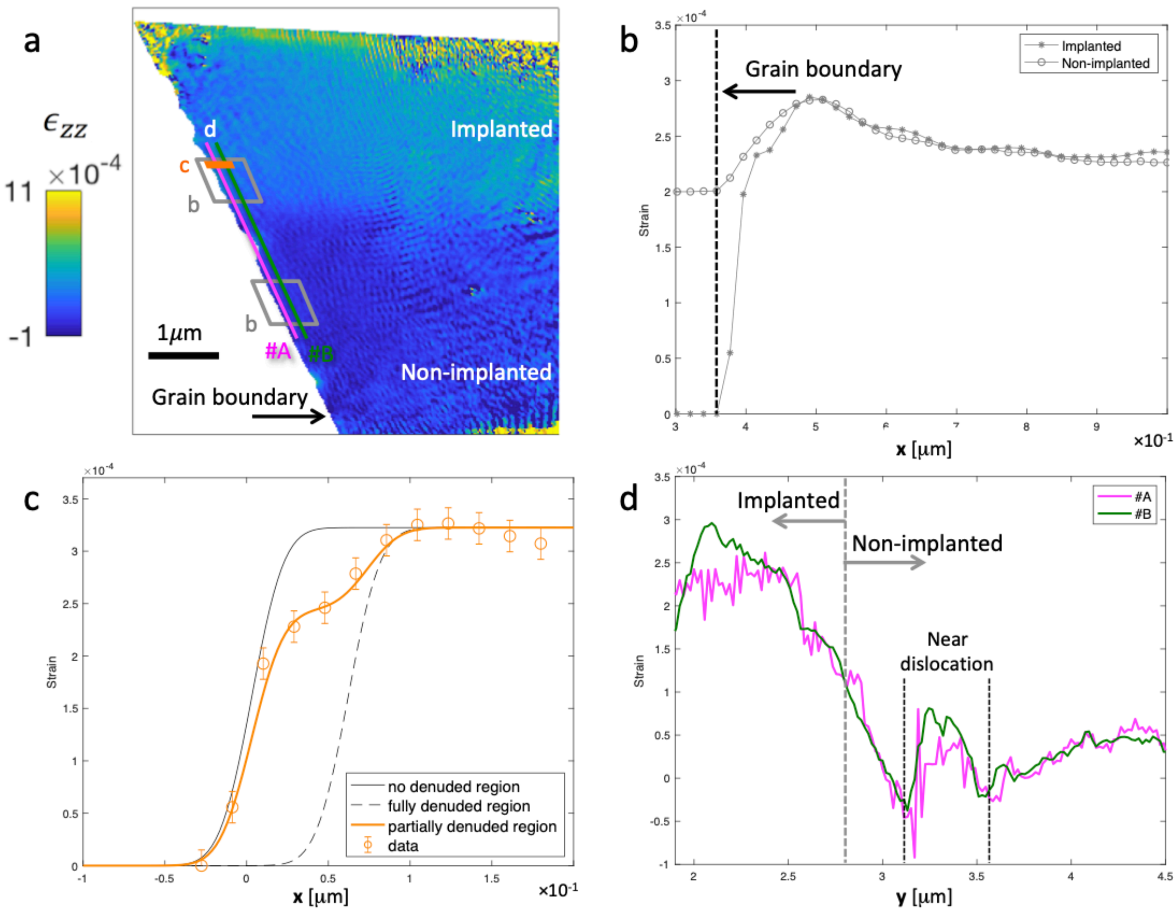

Figure 10 – Dislocation phase field analysis

Simulation details

Simulations of the phase variation around dislocations were carried out using the expressions for the displacement field associated with a triangular dislocation loop proposed by Barnett et al.[9,10] The sample was modeled as elastically isotropic with a Poisson ratio of 0.28.[11,12] This is appropriate since tungsten is almost perfectly isotropic, and the changes in the elastic constants of tungsten caused by 1% rhenium alloying and ion-implantation are small.[13,14] The crystal orientation determined from Laue diffraction was assigned to the model, where the sample **x'**, **y'** and **z'**-axes correspond to the following crystal directions: **x**: [-0.706 0.680 0.197], **y**: [0.125 -0.153 0.980] and **z**: [0.697 0.716 0.026]. Changes in the dislocation strain field at the sample surfaces, due to the traction free boundary condition, were not accounted for since the effects of surface relaxation only extend tens of nanometers into the sample[15] and, in the current sample, FIB-fabrication-induced strains dominate to this depth.

Based on the 3D reconstruction of the sample volume, dislocation #1 (observed in the implanted layer and presented in the main text in Fig. 4) was modeled as two partial dislocations, as two clearly distinct cores can be identified. Dislocation #2 (observed in the un-implanted region and presented hereafter) was modeled as a single dislocation, since here only one compact core is visible. The dislocation line direction was estimated based on the 3D reconstructed sample volume, and the dislocation lines were extended 5 microns above and below the sample mid plane. These dislocation segments were then linked to a remote closure point to form dislocation triangles. The two partial dislocation in dislocation #1 were assigned Burgers vector $\mathbf{b}_v$ = a/4 [-1 -1 1], while dislocation #2 was assigned $\mathbf{b}_v$ = a/2 [1 1 -1], where a is the lattice constant for tungsten.

The displacement field, $\mathbf{u_d}(\mathbf{r})$, due to dislocations was computed on the mid-plane of the sample. The phase variation anticipated in the reconstruction of the (220) crystal reflection, $\phi_{d,220}(\mathbf{r})$, was computed as $\phi_{d,220}(\mathbf{r}) = \mathbf{Q}_{220} . \mathbf{u_d}(\mathbf{r})$, where $\mathbf{Q}_{220}$ is the Bragg vector associated with the (220) reflection. The resulting phase map is in good agreement with the measured one.

Figure caption

(a) Zoomed-in 3D iso-surface density plots of the dislocation #2 highlighted by a white rectangle in the implanted layer, shown in Fig. 4(b). The presence of the dislocation appears as a void in the 3D density volume. (b) 2D cross-section map of the ϕ_{220} reconstructed phase, shown in the (**x**, **y**) plane in the vicinity of the dislocation. Local phase ramps, which are superimposing to the dislocation-induced phase contribution, need to be removed before further analysis. (c) Phase map to retrieve from (b) in order to extract the dislocation phase field shown in (d). The upper and lower parts of the phase map in (c) present two different linear phase gradients, corresponding to two slightly different lattice plane rotations. (d) Phase map obtained by subtracting (c) to ϕ_{220} (b). This should correspond to the dislocation contribution alone. (e) Estimated phase variation $\phi_{d,220}$ resulting from simulation. The phase color map in radians and the length scale bars are indicated in the figure.

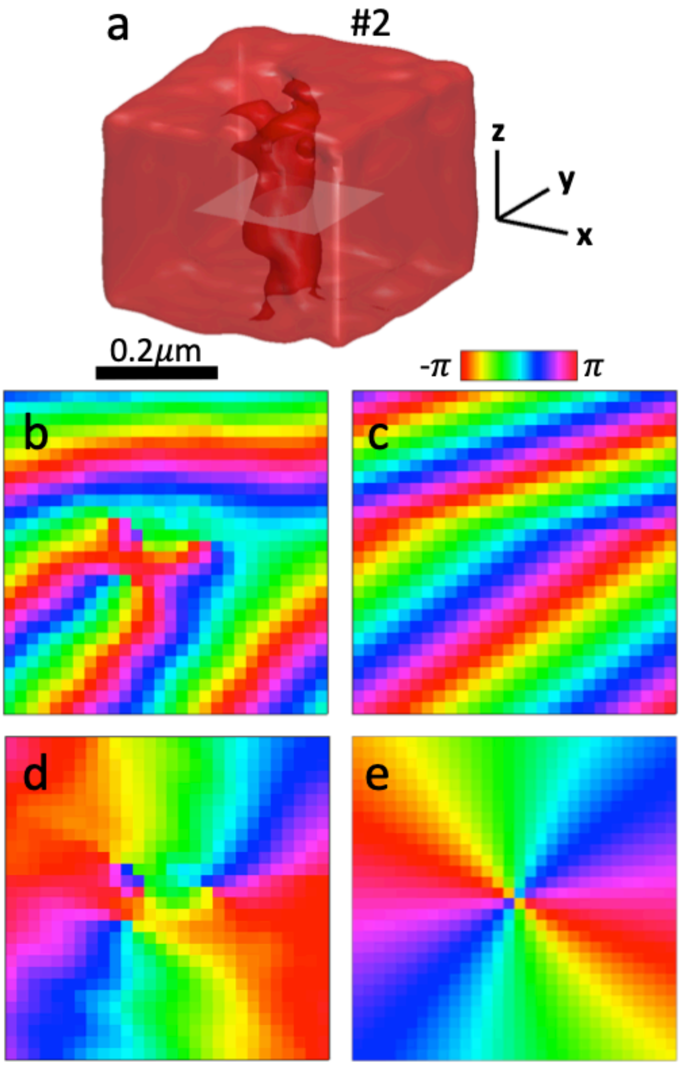
a
#2
z
y
x
0.2μm
-π
π
b
c
d
e

Figure 11 – 3D strain and tilts. Same as Fig. 4(a-f) from the main text, without the marked areas. The color scales are indicated on the plots.

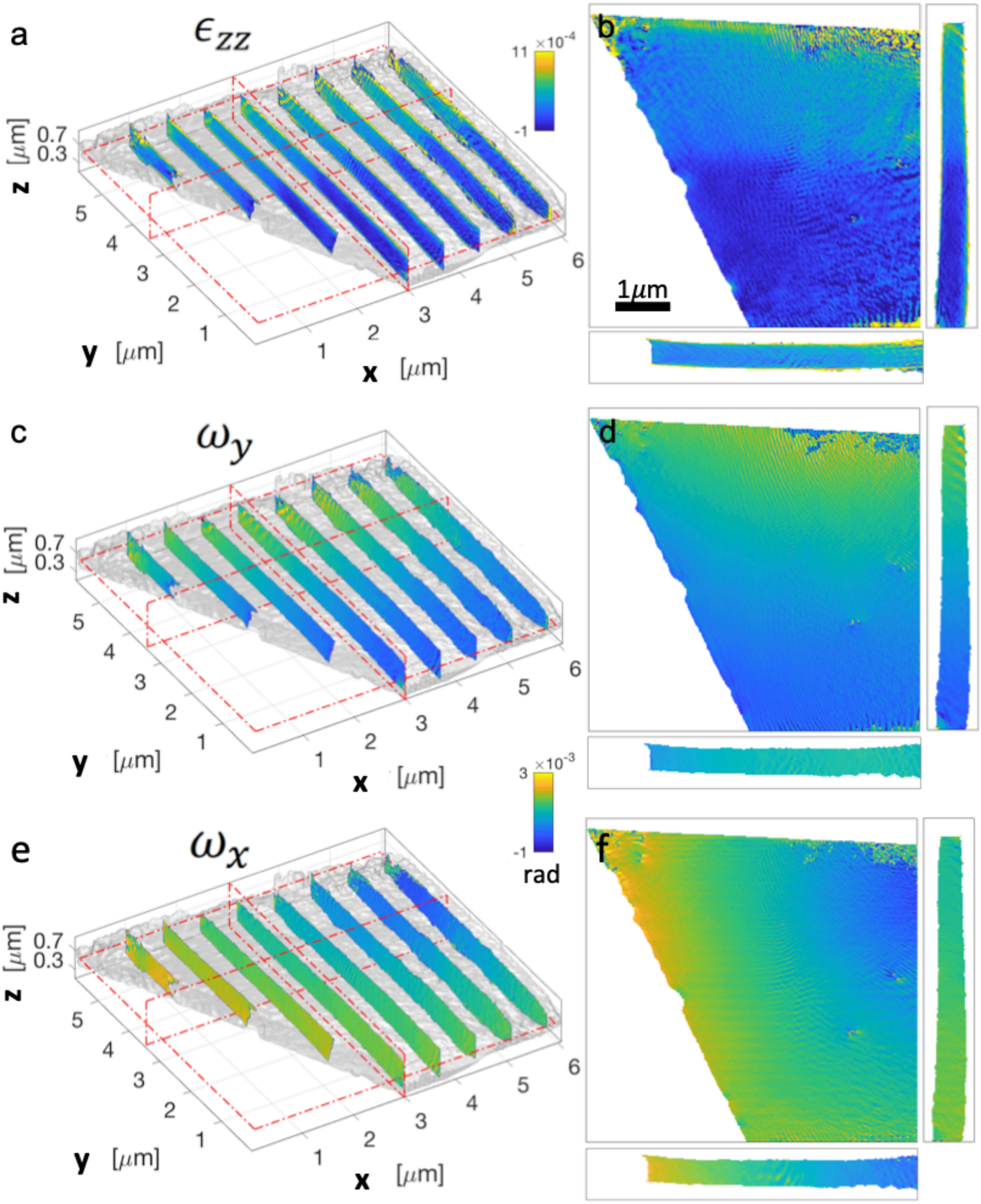

Table 1 – Ion energies and fluences. This table summarizes the ion fluences and energies used for the helium ion implantation.

Ion energy (MeV)	Fluence (ions/cm^2)
0.05	$1.50.10^{14}$
0.1	$2.50.10^{14}$
0.2	$3.50.10^{14}$
0.3	$1.50.10^{14}$
0.4	$4.00.10^{14}$
0.6	$4.75.10^{14}$
0.8	$4.50.10^{14}$
1.0	$4.75.10^{14}$
1.2	$4.75.10^{14}$
1.4	$5.00.10^{14}$
1.6	$5.50.10^{14}$
1.8	$5.50.10^{14}$

Supplementary references

1. van Heel, M. & Schatz, M. Fourier shell correlation threshold criteria. *J. Struct. Biol.* **151**, 250–262 (2005).
2. Wilkinson, A. J. & Britton, T. Ben. Strains, planes, and EBSD in materials science. *Mater. Today* **15**, 366–376 (2012).
3. Drouin, D. *et al.* CASINO V2.42: a fast and easy-to-use modeling tool for scanning electron microscopy and microanalysis users. *Scanning* **29**, 92–101 (2007).
4. Liu, W. *et al.* The three-dimensional X-ray crystal microscope: A new tool for materials characterization. *Metall. Mater. Trans. A* **35**, 1963–1967 (2004).
5. Hofmann, F., Eve, S., Belnoue, J., Micha, J.-S. & Korsunsky, A. M. Analysis of strain error sources in micro-beam Laue diffraction. *Nucl. Instrum. Methods Phys. Res. Sect. Accel. Spectrometers Detect. Assoc. Equip.* **660**, 130–137 (2011).
6. Hofmann, F. *et al.* X-ray micro-beam characterization of lattice rotations and distortions due to an individual dislocation. *Nat. Commun.* **4**, 2774 (2013).
7. Larson, B. C., Yang, W., Ice, G. E., Budai, J. D. & Tischler, J. Z. Three-dimensional X-ray structural microscopy with submicrometre resolution. *Nature* **415**, 887–890 (2002).
8. Larson, B. C. & Levine, L. E. Submicrometre-resolution polychromatic three-dimensional X-ray microscopy. *J. Appl. Crystallogr.* **46**, 153–164 (2013).
9. Barnett, D. M. The displacement field of a triangular dislocation loop. *Philos. Mag. A* **51**, 383–387 (1985).
10. Barnett, D. M. & Balluffi, R. W. The displacement field of a triangular dislocation loop–a correction with commentary. *Philos. Mag. Lett.* **87**, 943–944 (2007).
11. Bolef, D. I. & De Klerk, J. Elastic Constants of Single-Crystal Mo and W between 77° and 500°K. *J. Appl. Phys.* **33**, 2311–2314 (1962).
12. Featherston, F. H. & Neighbours, J. R. Elastic Constants of Tantalum, Tungsten, and Molybdenum. *Phys. Rev.* **130**, 1324–1333 (1963).
13. Duncan, R. A. *et al.* Increase in elastic anisotropy of single crystal tungsten upon He-ion implantation measured with laser-generated surface acoustic waves. *Appl. Phys. Lett.* **109**, 151906 (2016).
14. Hofmann, F. *et al.* Lattice swelling and modulus change in a helium-implanted tungsten alloy: X-ray micro-diffraction, surface acoustic wave measurements, and multiscale modelling. *Acta Mater.* **89**, 352–363 (2015).
15. Yoffe, E. H. A dislocation at a free surface. *Philos. Mag. J. Theor. Exp. Appl. Phys.* **6**, 1147–1155 (1961).
16. Ziegler, J. F., Ziegler, M. D. & Biersack, J. P. SRIM – The stopping and range of ions in matter (2010). *Nucl. Instrum. Methods Phys. Res. Sect. B Beam Interact. Mater. At.* **268**, 1818–1823 (2010).
17. Das, S., Liu, W., Xu, R. & Hofmann, F. Helium-implantation-induced lattice strains and defects in tungsten probed by X-ray micro-diffraction. *Mater. Des.* **160**, 1226–1237 (2018).
18. Rasch, K.-D., Siegel, R. W. & Schultz, H. Quenching and recovery investigations of vacancies in tungsten. *Philos. Mag. A* **41**, 91–117 (1980).
19. Debelle, A., Barthe, M. F. & Sauvage, T. First temperature stage evolution of irradiation-induced defects in tungsten studied by positron annihilation spectroscopy. *J. Nucl. Mater.* **376**, 216–221 (2008).
20. Nguyen-Manh, D., Horsfield, A. P. & Dudarev, S. L. Self-interstitial atom defects in bcc transition metals: Group-specific trends. *Phys. Rev. B* **73**, 020101 (2006).
21. Sandoval, L., Perez, D., Uberuaga, B. P. & Voter, A. F. Competing Kinetics and He

Bubble Morphology in W. *Phys. Rev. Lett.* **114**, 105502 (2015).
