# Supplementary material for: Revealing nano-scale lattice distortions in implanted material with 3D Bragg ptychography
Source: Nat Commun. 2021 Dec 3;12:7059. doi: 10.1038/s41467-021-27224-5 (PMC8642407; doi:10.1038/s41467-021-27224-5)
Supplement: Supplementary file 3 — Description of Additional Supplementary Files [file 41467_2021_27224_MOESM3_ESM.docx]

Description of Additional Supplementary Files:

**Supplementary Movie 1: 3D isosurface of the reconstructed object showing cross-sections of the extracted strain with cut-out.** All necessary scale bars (corresponding to 500 nm) and angular colour scales in radian are indicated on the plots.

**Supplementary Movie 2: 3D strain and tilts revealed in the x-y plane of the He-implanted polycrystalline tungsten foil with up-sampling of the angular positions and probe retrieval.** Cross-sections of the ε_zz_, ω_y_, ω_x_ maps are shown in the **x**-**y** plane for all values of **z** within the object array. All necessary scale bars (corresponding to 500 nm) and angular colour scales in radian are indicated on the plots.

**Supplementary Movie 3: 3D strain and tilts revealed in the y-z plane of the He-implanted polycrystalline tungsten foil with up-sampling of the angular positions and probe retrieval.** Cross-sections of the ε_zz_, ω_y_, ω_x_ maps are shown in the **y**-**z** plane for all values of **x** within the object array. All necessary scale bars (corresponding to 500 nm) and angular colour scales in radian are indicated on the plots.
